# Proteinuria is a Prognostic Marker for Cardiovascular Mortality: NIPPON DATA 80, 1980-1999

**DOI:** 10.2188/jea.15.146

**Published:** 2005-08-30

**Authors:** Shinichi Tanihara, Takehito Hayakawa, Izumi Oki, Yosikazu Nakamura, Kiyomi Sakata, Akira Okayama, Yasuyuki Fujita, Hirotsugu Ueshima

**Affiliations:** 1 Department of Public Health, School of Medicine, Shimane University.; 2 Department of Public Health, Jichi Medical School.; 3 Department of Hygiene and Preventive Medicine, Iwate Medical University School of Medicine.; 4 Department of Preventive Cardiology, National Cardiovascular Center.; 5 Department of Health Science, Shiga University of Medical Science.

**Keywords:** Proteinuria, Cardiovascular Diseases, Cohort Studies, Japan, Mortality

## Abstract

BACKGROUND: Proteinuria has been considered to be a prognostic marker for persons with diabetes mellitus, but only a limited number of studies about the relationship between proteinuria and mortality among general population has been available.

METHODS: The subjects were 10,897 individuals who participated in the National Cardiovascular Survey conducted in 1980 and who were aged 30 years or older living in 300 districts that had been randomly selected throughout Japan. The vital records were confirmed in 1999 and 7,203 subjects (3,180 males and 4,023 females) without a history of hypertension, stroke, heart disease, renal disease, or diabetes mellitus at the start of the study were investigated.

RESULTS: There were 126,825 person-years of follow-up. During the observed period of time, 371 died of cardiovascular causes, including 171 stroke deaths and 74 coronary deaths. The risk of proteinuria for cardiovascular mortality was greater than unity for those with a normal serum creatinine level, after adjusting for age and other cardiovascular disease risk factors.

CONCLUSIONS: When contrasted with other cardiovascular disease risk factors, urinary protein is an independent risk factor for cardiovascular death among the Japanese population.

Proteinuria or urinary albumin has been considered to be a prognostic marker for patients with non-insulin-dependent diabetes mellitus^1^^-^^14^ and those with hypertension^10^^,^^15^^-^^18^ or acute myocardial infarction.^19^ However, in general population, knowledge about the relationship between proteinuria and mortality is limited.^14^ Proteinuria has been a predictor of mortality in subjects aged 65-79 years^20^ but not among those 80 years and older.^21^ Urinary albumin is related to risk factors for cardiovascular diseases,^15^^,^^22^ but in only a few studies has diabetes mellitus or the history of renal diseases been assessed.^23^ In addition, most of these studies have been conducted in European countries and in the United States. Studies about the relationship between proteinuria and mortality among non-whites are also limited.^8^^,^^10^ In this study, we describe the relationship between proteinuria at baseline and death from cardiovascular disease in a nationally representative cohort of the Japanese population.

## METHODS

### Study Subjects

The subjects were the participants of The National Cardiovascular Survey of 1980.^24^ The procedures of the 1980 National Cardiovascular Survey have already been described.^24^^-^^26^ In 1980, all household members aged 30 years or older living in 300 districts randomly selected throughout Japan were counted. There were 13,771 eligible subjects, among which 10,897 (79.4%) participated in the national survey.

### Baseline Information

Using a standard color tone table, the urine dipstick test for protein was carried out and assessed as: none, trace, +, or ++. Blood samples were obtained during a non-fasting state and the time (in hours) between the last meal and the time of sample collection was recorded. Creatinine was measured by using the Jaffe method. Blood sugar was measured by the Neocaproine-copper method. A Technicon SMA 12-60 (Technicon Instruments, Tarrytown, NY) was used for these measurements. Hyperglycemia was defined as a blood sugar level of 7.7mmol/L or greater when the time from the last meal before blood sampling was two hours or longer and/or 11.1 mmol/L or greater when the elapsed time between the last meal and sampling was less than two hours.

A self-administered questionnaire was used to obtain information on history of gout, hypertension, diabetes mellitus, stroke, heart disease, renal disease, smoking, and alcohol drinking habits. The use of antihypertensive agents was asked to persons with a history of hypertension, although information on specific types of drugs was not obtained. For alcohol drinking habits, the subjects were asked whether they “never drink”; “used to drink”; “occasionally drink”; or “drink daily”. For smoking habits, similar questions were posed: whether they “never smoked”; “used to smoke”; or “currently smoke”. Both habits were also separated into “current” and “non-current” status. Current drinking included both occasional and daily drinking.

A standard sphygmomanometer was used to measure blood pressure; and the first and the 5th Korotkoff’s sounds were recorded as the systolic and diastolic blood pressures. The definition of hypertension was a systolic blood pressure of 160 mmHg or higher and/or a diastolic blood pressure of 95 mmHg or higher. Body weight was measured with light clothes and no shoe.

### Follow-up

In 1994, a follow-up study was conducted with the participants of this survey, which was called the ‘National Integrated Projects for Prospective Observation of Non-Communicable Diseases and the Trend in the Aged’ (NIPPON DATA 80).^25^^,^^26^

A total of 10,546 subjects for whom complete information on age, sex, and blood pressure from the 1980 data set was available made up a cohort. The vital status of these subjects was determined by reviewing the resident registry system of 1994 and 1999. The underlying cause of death of those who died during the follow-up period was obtained from death certificates and coded according to the International Classification of Diseases, 9th revision (ICD9) for the period between 1980 and 1994; and 10th revision (ICD10) for the period between 1995 and 1999. Deaths from stroke (ICD9: 430-438, ICD10: I60-I69), cardiovascular diseases (ICD9: 390-459, ICD10: I00-I99), and cancer (ICD9: 140-208, ICD10: C00-C97) were defined as ICD9 or ICD10 codes.

After a follow-up that lasted for 19 years, 908 were lost to follow-up and 759 were excluded because of missing data for potential confounders. For the survival analysis, an additional 1,676 persons with histories of hypertension, stroke, heart disease, renal disease, or diabetes mellitus were excluded because proteinuria or urinary albumin is a predictor of cardiovascular disease mortality for persons with diabetes^9^^,^^11^^,^^12^ or hypertension.^15^^-^^18^ Finally, 7,203 subjects (3,180 males and 4,023 females) were selected for the study.

### Statistical Analysis

Cox’s proportional hazards regression models were used to examine the relationship between proteinuria and cardiovascular mortality. First, proteinuria was considered as a categorical variable and hazard ratios (HR) were calculated for each category (trace, +, ++ and more). Next, proteinuria was diagnosed when a subject showed trace, +, or ++ on the urine dipstick test and considered as a dichotomous variable. The subjects were also divided into three categories according to body mass index (BMI, kg/m^2^) as follows: “lean” (≤20.0), “standard” (20.1-24.9), and “obese” (25+); and classified according to serum cholesterol (mmol/L) levels as follows: “low” (<4.1), “standard” (4.1-6.1), and “high” (6.2+). The serum creatinine level (*μ*mol/L) was rated separately for men and women.^27^ Male subjects were divided into three categories: “low” (<97), “standard” (97-105), and “high” (106+). For women, the criteria were set as follows: “low” (<71); “standard” (71-79); and “high” (80+).^27^

For comparative purposes, the hazard ratios were calculated with adjustments for age only (grouped by 10-year increments), and with adjustment for age, hypertension (yes/no), hyperglycemia (yes/no), current smoking status (yes/no), current drinking status (yes/no), BMI (lean, standard, or obese), serum cholesterol level (low, standard, or high). To assess the interaction between urinary protein and serum creatinine level, multivariate analyses stratified by the three levels of serum creatinine were conducted. Analyses were performed separately for men and women.

All the analyses was performed with SAS^®^ software (Version 8.2 SAS Institute, Cary, NC). Two-sided values where p < 0.05 were considered statistically significant.

## RESULTS

Table 1 shows the number of deaths according to underlying cause of death stratified by baseline proteinuria level. The 19-year follow-up lasted for 126,825 person-years, during which 1,179 subjects died. Of these, 371 died of cardiovascular causes, including 171 stroke deaths and 71 coronary deaths, and 831 of non-cardiovascular deaths, including 393 who died of cancer and 70 as a result of accidents and injuries. The crude cardiovascular mortality increased with the level of proteinuria for both men and women.

**Table 1.** The number of deaths according to underlying cause stratified by baseline proteinuria level.

Causes of death	baseline proteinuria level				

	total	negative	trace	+	++ and more
	Male				
cardiovascular	197	175	13	4	5
cancer	237	224	8	4	1
non-cardiovascular, non-cancer	223	203	14	6	0
All-cause	657	602	35	14	6
	(n=3180)	(n=2994)	(n=121)	(n=50)	(n=15)

	Female				
cardiovascular	174	154	9	9	2
cancer	156	145	7	4	0
non-cardiovascular, non-cancer	192	178	10	3	1
All-cause	522	477	26	16	3
	(n=4023)	(n=3804)	(n=150)	(n=57)	(n=12)

Among the 395 male subjects lost in follow-up, the number of subjects with proteinuria (including trace or more) was 33 (8.4%), as for trace was 16 (4.1%), + was 15 (3.8%), and ++ and more was 2 (0.5%). Among the 513 female subjects lost in follow-up, the number of subjects with proteinuria (including trace or more) was 48(9.4%), as for trace was 26 (5.1%), + was 20 (3.9%), and ++ and more was 2 (0.4%). Among subjects lost in follow-up, the proportion of subjects with proteinuria was higher than that of subjects completed the follow-up for both men and women.

Table 2 shows the baseline characteristics for subjects with and without proteinuria. For both men and women, the mean age, body mass index, systolic blood pressure, diastolic blood pressure, serum total cholesterol and glucose levels were significantly greater in the subjects with proteinuria. The proportions of subjects with a high serum cholesterol level, obesity, hypertension, or hyperglycemia were significantly greater in the group with proteinuria for both men and women.

**Table 2.** Baseline characteristics of study subjects in 1980 NIPPON DATA, 3180 men and 4203 women aged 30-91 years.

Proteinuria	Male				Female			
	
	Negative		Positive*		Negative		Positive*	
	(n=2994)		(n=186)		(n=3804)		(n=219)	

Characteristics	mean	SD	mean	SD	mean	SD	mean	SD

Age (year)	48.6	12.6	52.6	14.0	48.9	12.7	50.2	14.4
Body mass index (kg/m^2^)	22.4	3.0	23.2	3.2	22.6	3.2	23.3	4.1
Systoric blood pressure (mmHg)	135.4	19.1	141.9	22.3	130.0	18.7	137.8	23.0
Diastolic blood pressure (mmHg)	82.3	11.5	85.3	13.8	78.0	11.0	81.4	12.8
Serum total cholesterol (mmol/L)	4.9	0.9	5.0	0.9	4.9	0.9	5.0	0.9
Serum creatinine (*μ*mol/L)	81.3	14.1	84.9	17.7	80.0	17.7	48.9	12.7
Serum glucose (mmol/L)	7.1	1.7	7.5	2.2	7.0	1.8	7.1	1.8
Follow-up time (year)	17.4	3.9	16.3	5.2	17.9	3.3	16.9	4.8

Current smoker	1915	(64.0%)	123	(66.1%)	328	(8.6%)	24	(11.0%)
Current drinker	2264	(75.6%)	136	(73.1%)	783	(20.6%)	44	(20.1%)
High serum cholesterol	161	(5.4%)	18	(9.7%)	281	(7.4%)	25	(11.4%)
Low serum cholesterol	646	(21.6%)	33	(17.7%)	747	(19.6%)	32	(14.6%)
Leanness (BMI<20)	598	(20.0%)	32	(17.2%)	766	(20.1%)	53	(24.2%)
Obesity (BMI>25)	526	(17.6%)	49	(26.3%)	745	(19.6%)	67	(30.6%)
Hypertension	533	(17.8%)	54	(29.0%)	397	(10.4%)	57	(26.0%)
Hyperglycemia	464	(15.5%)	51	(27.4%)	617	(16.2%)	50	(22.8%)
Creatinine level								
High	1716	(57.3%)	96	(51.6%)	942	(24.8%)	44	(20.1%)
Standard	678	(22.6%)	46	(24.7%)	1311	(34.5%)	65	(29.7%)
Low	600	(20.0%)	44	(23.7%)	1551	(40.8%)	110	(50.2%)

* includes trace, +, ++ and more.

Hyperglycemia was defined as a blood sugar level of 7.7 mmol/L or greater when the time from the last meal before blood sampling was two hours or longer and/or11.1mmol/L or greater when the elapsed time between the last meal and sampling was less than two hours.

High serum cholesterol was defined as a serum cholesterol level of 6.2 mmol/L or greater. Low serum cholesterol was defined when a serum cholesterol level was less than 4.1 mmol/L.

The definition of hypertension was a systolic blood pressure of 160 mmHg or greater and/or a diastolic blood pressure of 95 mmHg or greater.

The serum creatinine level (*μ*mol/L) was rated separately for men “low” (serum creatinine<97), “standard” (97-105), and“high”(106+) and women“low”(<71); “standard”(71-79); and “high”(80+).

Table 3 shows the hazard ratios of proteinuria with all cause and cardiovascular mortality by sex. When examined by using the Cox proportional hazards model adjusted for age only, female subjects with “+” proteinuria significantly associated with all cause mortality. The hazard ratios for male subjects with “++ and more” proteinuria were higher than unity without significance. Proteinuria including trace, +, ++ and more increased the risk with statistical significance with all cause mortality for both men (HR=1.58, 95% confidence interval [CI]: 1.20-2.08) and women (HR=1.75, 95% CI: 1.29-2.38). From the results of multivariate analysis, the hazard ratios of proteinuria with all cause mortality were similar to the models adjusted for age only. The hazard ratios for male subjects with proteinuria including trace, +, ++ and more were higher than unity without significance.

**Table 3.** Hazard ratios of proteinuria for all cause and cardiovascular mortality.

	All cause mortality		Cardiovascular mortality	
	
	Age-adjusted	Multivariate	Age-adjusted	Multivariate
	hazard ratio (95% CI)	hazard ratio (95% CI)	hazard ratio (95% CI)	hazard ratio (95% CI)
	Male			
negative	1.00 (reference)	1.00 (reference)	1.00 (reference)	1.00 (reference)
trace	1.13 (0.77-1.65)	1.13 (0.77-1.65)	1.48 (0.80-2.73)	1.44 (0.78-2.67)
+	0.72 (0.38-1.34)	0.67 (0.35-1.26)	0.46 (0.12-1.87)	0.35 (0.09-1.44)
++ and more	2.07 (0.86-4.98)	1.76 (0.72-4.29)	6.21 (2.29-16.80)	4.20 (1.50-11.72)

p-value for trend	0.1443	0.2083	0.0062	0.0467

trace,+,++ and more*	1.58 (1.20-2.08)	1.22 (0.92-1.61)	2.17 (1.39-3.38)	1.49 (0.95-2.34)

	Female			
negative	1.00 (reference)	1.00 (reference)	1.00 (reference)	1.00 (reference)
trace	1.50 (0.98-2.30)	1.48 (0.96-2.28)	1.56 (0.73-3.34)	1.61 (0.75-3.47)
+	1.97 (1.08-3.58)	1.91 (1.04-3.52)	3.42 (1.51-7.75)	3.08 (1.31-7.26)
++ and more*	1.30 (0.42-4.05)	1.19 (0.38-3.74)	2.27 (0.56-9.19)	2.01 (0.48-8.31)

p-value for trend	0.0005	0.0016	0.0002	0.0016

trace,+,++ and more*	1.75 (1.29-2.38)	1.74 (1.27-2.38)	2.41 (1.51-3.84)	2.21 (1.36-3.59)

Age, smoking, drinking, serum cholesterol, hyperglycemia, leanness, obesity, and hypertension were adjusted in multivariate analysis.

* :regrouped

CI: confidence interval

The hazard ratios for cardiovascular mortality increased with the level of proteinuria for both men and women when examined by using the Cox proportional hazards model adjusted for age only. Male subjects with “++” proteinuria and female subjects with “+” proteinuria significantly associated with cardiovascular mortality. The hazard ratios for subjects with “trace” proteinuria were higher than unity for both men and women without significance. Proteinuria including trace, +, ++ and more increased the risk with statistical significance with cardiovascular mortality for both men (HR=2.17, 95% CI: 1.39-3.38) and women (HR=2.41, 95% CI: 1.51-3.84).

From the results of multivariate analysis, the hazard ratios of proteinuria with cardiovascular mortality were similar to the models adjusted for age only. The hazard ratios for female subjects with proteinuria including trace, +, ++ and more were higher than unity with statistical significance. Those for male were higher than unity although they were not statistically significant. Even when the deceased or those lost in the first three years of follow-up were excluded, the risk of proteinuria for cardiovascular mortality among women was significantly higher than unity (HR=2.04, 95% CI: 1.18-3.54). The hazard ratios for male subjects were not statistically significant (HR=1.21, 95% CI: 0.73-2.01).

Table 4 shows the results of stratified analyses by the three levels of serum creatinine. Some of the results from the stratified analysis by serum creatinine level were different from those of age only adjusted models and multivariate analysis. For males, the risk of proteinuria for cardiovascular mortality was significantly higher than unity in the group with standard serum creatinine level. For female subjects, the risk of proteinuria was significantly higher than unity in the group with high creatinine level.

**Table 4.** Stratified analysis by serum creatinine level for proteinuria for cardiovascular mortality.

serum creatinine level (*μ*mol/L)	n	Proteinuria positive* (%)	Cardiovascular death (%)	Hazard ratio	(95% CI)
	Male (n=3180)				
106+	644	96 (14.9%)	60 (9.3%)	1.07	(0.45 -2.57)
97-105	724	44 ( 6.1%)	42 (5.8%)	4.07	(1.80 -9.20)
-96	1812	46 ( 2.5%)	9 (0.5%)	0.84	(0.36 -1.93)

	Female (n=4023)				
80+	1661	44 ( 2.6%)	104 (6.3%)	2.36	(1.33 -4.19)
71-79	1376	65 ( 4.7%)	42 (3.1%)	2.41	(0.77 -7.54)
-70	986	110 (11.2%)	28 (2.8%)	1.03	(0.12 -9.17)

Adjusted for age, smoking, drinking, serum cholesterol, hyperglycemia, leanness, obesity, and hypertension.

Proteinuria includes trace,+,++ and more.

## DISCUSSION

Our results indicated that proteinuria is related to an increased risk of death from cardiovascular disease among persons with no history of diabetes mellitus, hypertension, acute myocardial infarction, or stroke. Prospective data on proteinuria and mortality among the general population are limited.^14^ A number of prospective epidemiologic studies have reported that proteinuria or urinary albumin is a predictor of death for those with diabetes^1^^-^^14^ and hypertension.^10^^,^^15^^-^^18^ Most of the prospective studies on urinary protein or albumin and mortality were conducted in European countries^2^^,^^5^^,^^6^^,^^9^^,^^12^^,^^14^^,^^15^ and in the United States.^3^^,^^11^^,^^16^ The results of our study are unique because the study subjects were composed of a representative cohort of the Japanese population.

The hazard ratios for cardiovascular mortality increased with the level of proteinuria. The level of proteinuria was proportional to the risk of mortality.^16^ In this study, the positive sign of urinary protein is defined according to the urinary dipstick test result (as trace, +, ++ and more). Although without significance, the hazard ratios for cardiovascular mortality in subjects with “trace” proteinuria were higher than unity for both men and women. It is consistent with studies reporting that even lesser degrees of albuminuria predict cardiovascular events even after subjects with dipstick-positive (i.e., less than or equal to +) proteinuria had been excluded.^11^^,^^13^

Our results showed that urinary protein, measured by dipstick methods, is an independent risk factor for cardiovascular death. In the most of studies, urinary protein was measured with a dipstick and the positive sign for proteinuria was defined when the test result was + or more,^11^^,^^16^ 300+mg/24h, 12 or >30mg/dL.^20^ Measuring urinary protein with a dipstick is a useful screening test because it is very simple and inexpensive. Finding the optimal cut-off point in the urinary dipstick test requires further consideration.

There are some limitations to be considered in this study. For example, potentially important confounding factors, such as post-menopausal status^14^ and waist and hip measurements^28^ were not obtained at the initial survey.

Among subjects lost in follow-up, the proportion of subjects with proteinuria was higher than subjects selected for the analysis. The results of this study underestimated true relation between mortality and urine protein because it is presumable that subjects lost in follow-up have higher all cause and cardiovascular mortality. It means that the direction of bias produced by subjects lost in follow-up is toward null value. The observed hazard ratios are probably closer to the null than what it would be if the subjects lost in follow-up were absent.

Urinary protein was measured only once using an available urine sample so that it is possible that it may be misclassified in reading the results. It is also true that urinary protein is determined at the baseline before the survival or cause of death of the surveyed subjects becomes known through follow-up studies; and it is judged that any misclassification, if it occurs, is non-differential. If so, the effect of the risk factor that has been computed must be smaller than the real value and has no bearing on the conclusion of the present study, i.e., urinary protein is an independent risk factor of mortality from cardiovascular diseases.

Microalbuminuria correlates with cardiovascular autonomic dysfunction and insulin resistance in type 2 diabetic patients.^29^ In hypertensive subjects, the inflamatory injury in the kidney structures consequent to that of myocardial infarction causes a greater albumin leak.^30^ However, precise underlying pathophysiologic mechanisms of the association between proteinuria and unfavorable cardiovascular outcome among persons with no history of diabetes mellitus, hypertension, acute myocardial infarction, or stroke have not been totally given.

The results of the analysis that excluded subjects deceased or lost in the first three years of follow-up and the stratified analysis by serum creatinine level were different with men and women. For males, the risk of proteinuria for cardiovascular mortality was significantly higher than unity in the group with standard serum creatinine level. For female subjects, the risk of proteinuria was significantly higher than unity in the group with high creatinine level. It is reported that a possible difference in the mechanism or significance of urinary albumin excretion between both genders.^31^ Future studies on proteinuria should take factors related sex, menopausal status for example, into account.

In conclusion, urinary protein is an independent risk factor for cardiovascular death among the Japanese population especially in relation to their medical histories, blood pressure status and blood sugar level. Measuring urinary protein by the dipstick method is useful in locating persons with a high risk for cardiovascular mortality because it is simple and easy to conduct during a mass screening.

## APPENDIX

### List of the NIPPON DATA80 Research group

NIPPON DATA80: “National Integrated Projects for Prospective Observation of Non-communicable Diseases And its Trends in the Aged”

**Chairman:** Hirotsugu Ueshima (Department of Health Science, Shiga University of Medical Science, Otsu, Shiga)

**Consultant:** Osamu Iimura (Hokkaido JR Sapporo Hospital, Sapporo, Hokkaido), Teruo Omae (National Cardiovascular Center, Suita, Osaka), Kazuo Ueda (School of Health Science, Kyushu University, Fukuoka, Fukuoka), Hiroshi Yanagawa (Saitama Prefectural University, Koshigaya, Saitama)

**Research Member:** Akira Okayama (Preventive Cardiology National Cardiovascular Disease Center, Suita, Osaka), Kazunori Kodama, Fumiyoshi Kasagi (Radiation Effects Research Foundation, Hiroshima, Hiroshima), Tomonori Okamura, Yoshikuni Kita (Department of Health Science, Shiga University of Medical Science, Otsu, Shiga), Shigeyuki Saito (Internal Medicine II, Cardiology and Nephrology, Sapporo Medical University School of Medicine, Sapporo, Hokkaido), Kiyomi Sakata (Department of Hygiene and Preventive Medicine, Iwate Medical University School of Medicine, Morioka, Iwate), Takehito Hayakawa, Shinichi Tanihara (Department of Public Health, School of Medicine, Shimane University, Izumo, Shimane), Yosikazu Nakamura (Department of Public Health, Jichi Medical School, Minami Kawachi, Tochigi), Hiroshi Horibe (Keisen Clinic, Akashi, Hyogo), Masumi Minowa (Department of Epidemiology, National Institute of Public Health, Wako, Saitama)

**Research Associate Member:** Toshihiro Takeuchi, Mitsuru Hasebe, Fumitsugu Kusano and members of 300 Public Health Centers in Japan, Katsuhiko Kawaminami (Department of Public Health Policy, National Institute of Public Health, Wako, Saitama), Sohel R. Choudhury, (Department of Community Medicine, School of Medical Sciences University Sains Malaysia), Yutaka Kiyohara (Department of Medicine and Clinical Science, Graduate School of Medical Sciences, Kyusyu University, Fukuoka, Fukuoka), Minoru Iida (Kansai University of Welfare Sciences, Osaka), Tsutomu Hashimoto (Wakayama Red Cross Blood Center, Wakayama, Wakayama), Atsushi Terao (Hikone Public Health Center, Hikone, Shiga), Koryo Sawai (The Japanese Association for Cerebro-cardiovascular Disease Control, Tokyo, Tokyo), Shigeo Shibata (Clinical Nutrition, Kagawa Nutrition University, Saitama)
